# Supplementary material for: OpenMM 7: Rapid development of high performance algorithms for molecular dynamics
Source: PLoS Comput Biol. 2017 Jul 26;13(7):e1005659. doi: 10.1371/journal.pcbi.1005659 (PMC5549999; doi:10.1371/journal.pcbi.1005659)
Supplement: S1 Source Code — (ZIP) [file pcbi.1005659.s002.zip › OpenMM-7.0.1-Source/docs/api-c++/generated/OpenMM.DrudeForce.html]

DrudeForce — OpenMM @OPENMM\_MAJOR\_VERSION@.@OPENMM\_MINOR\_VERSION@ documentation


# DrudeForce¶

*class* `OpenMM::``DrudeForce`¶
:   This class implements forces that are specific to Drude oscillators. There are two distinct forces it applies: an anisotropic harmonic force connecting each Drude particle to its parent particle; and a screened Coulomb interaction between specific pairs of dipoles. The latter is typically used between closely bonded particles whose Coulomb interaction would otherwise be fully excluded.

    To use this class, create a `DrudeForce` object, then call `addParticle()` once for each Drude particle in the `System` to define its parameters. After a particle has been added, you can modify its force field parameters by calling `setParticleParameters()`. This will have no effect on Contexts that already exist unless you call `updateParametersInContext()`. Likewise, call `addScreenedPair()` for each pair of dipoles (each dipole consisting of a Drude particle and its parent) that should be computed.

    Methods

    |  |  |
    | --- | --- |
    | `DrudeForce` | Create a `DrudeForce`. |
    | `getNumParticles` | Get the number of particles for which force field parameters have been defined. |
    | `getNumScreenedPairs` | Get the number of special interactions that should be calculated differently from other interactions. |
    | `addParticle` | Add a Drude particle to which forces should be applied. |
    | `getParticleParameters` | Get the parameters for a Drude particle. |
    | `setParticleParameters` | Set the parameters for a Drude particle. |
    | `addScreenedPair` | Add an interaction to the list of screened pairs. |
    | `getScreenedPairParameters` | Get the force field parameters for screened pair. |
    | `setScreenedPairParameters` | Set the force field parameters for screened pair. |
    | `updateParametersInContext` | Update the particle and screened pair parameters in a `Context` to match those stored in this `Force` object. |
    | `usesPeriodicBoundaryConditions` | Returns whether or not this force makes use of periodic boundary conditions. |

    `DrudeForce`()¶
    :   Create a `DrudeForce`.

    int `getNumParticles`() *const*¶
    :   Get the number of particles for which force field parameters have been defined.

    int `getNumScreenedPairs`() *const*¶
    :   Get the number of special interactions that should be calculated differently from other interactions.

    int `addParticle`(int *particle*, int *particle1*, int *particle2*, int *particle3*, int *particle4*, double *charge*, double *polarizability*, double *aniso12*, double *aniso34*)¶
    :   Add a Drude particle to which forces should be applied.

        |  |  |
        | --- | --- |
        | Parameters: |  |

        - **particle** – the index within the `System` of the Drude particle
        - **particle1** – the index within the `System` of the particle to which the Drude particle is attached
        - **particle2** – the index within the `System` of the second particle used for defining anisotropic polarizability. This may be set to -1, in which case aniso12 will be ignored.
        - **particle3** – the index within the `System` of the third particle used for defining anisotropic polarizability. This may be set to -1, in which case aniso34 will be ignored.
        - **particle4** – the index within the `System` of the fourth particle used for defining anisotropic polarizability. This may be set to -1, in which case aniso34 will be ignored.
        - **charge** – The charge on the Drude particle
        - **polarizability** – The isotropic polarizability
        - **aniso12** – The scale factor for the polarizability along the direction defined by particle1 and particle2
        - **aniso34** – The scale factor for the polarizability along the direction defined by particle3 and particle4

        |  |  |
        | --- | --- |
        | Returns: | the index of the particle that was added |

    void `getParticleParameters`(int *index*, int &*particle*, int &*particle1*, int &*particle2*, int &*particle3*, int &*particle4*, double &*charge*, double &*polarizability*, double &*aniso12*, double &*aniso34*) *const*¶
    :   Get the parameters for a Drude particle.

        |  |  |
        | --- | --- |
        | Parameters: |  |

        - **index** – the index of the Drude particle for which to get parameters
        - **particle** – [out] the index within the `System` of the Drude particle
        - **particle1** – [out] the index within the `System` of the particle to which the Drude particle is attached
        - **particle2** – [out] the index within the `System` of the second particle used for defining anisotropic polarizability. This may be set to -1, in which case aniso12 will be ignored.
        - **particle3** – [out] the index within the `System` of the third particle used for defining anisotropic polarizability. This may be set to -1, in which case aniso34 will be ignored.
        - **particle4** – [out] the index within the `System` of the fourth particle used for defining anisotropic polarizability. This may be set to -1, in which case aniso34 will be ignored.
        - **charge** – [out] The charge on the Drude particle
        - **polarizability** – [out] The isotropic polarizability
        - **aniso12** – [out] The scale factor for the polarizability along the direction defined by particle1 and particle2
        - **aniso34** – [out] The scale factor for the polarizability along the direction defined by particle3 and particle4

    void `setParticleParameters`(int *index*, int *particle*, int *particle1*, int *particle2*, int *particle3*, int *particle4*, double *charge*, double *polarizability*, double *aniso12*, double *aniso34*)¶
    :   Set the parameters for a Drude particle.

        |  |  |
        | --- | --- |
        | Parameters: |  |

        - **index** – the index of the Drude particle for which to set parameters
        - **particle** – the index within the `System` of the Drude particle
        - **particle1** – the index within the `System` of the particle to which the Drude particle is attached
        - **particle2** – the index within the `System` of the second particle used for defining anisotropic polarizability. This may be set to -1, in which case aniso12 will be ignored.
        - **particle3** – the index within the `System` of the third particle used for defining anisotropic polarizability. This may be set to -1, in which case aniso34 will be ignored.
        - **particle4** – the index within the `System` of the fourth particle used for defining anisotropic polarizability. This may be set to -1, in which case aniso34 will be ignored.
        - **charge** – The charge on the Drude particle
        - **polarizability** – The isotropic polarizability
        - **aniso12** – The scale factor for the polarizability along the direction defined by particle1 and particle2
        - **aniso34** – The scale factor for the polarizability along the direction defined by particle3 and particle4

    int `addScreenedPair`(int *particle1*, int *particle2*, double *thole*)¶
    :   Add an interaction to the list of screened pairs.

        |  |  |
        | --- | --- |
        | Parameters: |  |

        - **particle1** – the index within this `Force` of the first particle involved in the interaction
        - **particle2** – the index within this `Force` of the second particle involved in the interaction
        - **thole** – the Thole screening factor

        |  |  |
        | --- | --- |
        | Returns: | the index of the screenedPair that was added |

    void `getScreenedPairParameters`(int *index*, int &*particle1*, int &*particle2*, double &*thole*) *const*¶
    :   Get the force field parameters for screened pair.

        |  |  |
        | --- | --- |
        | Parameters: |  |

        - **index** – the index of the pair for which to get parameters
        - **particle1** – [out] the index within this `Force` of the first particle involved in the interaction
        - **particle2** – [out] the index within this `Force` of the second particle involved in the interaction
        - **thole** – [out] the Thole screening factor

    void `setScreenedPairParameters`(int *index*, int *particle1*, int *particle2*, double *thole*)¶
    :   Set the force field parameters for screened pair.

        |  |  |
        | --- | --- |
        | Parameters: |  |

        - **index** – the index of the pair for which to get parameters
        - **particle1** – the index within this `Force` of the first particle involved in the interaction
        - **particle2** – the index within this `Force` of the second particle involved in the interaction
        - **thole** – the Thole screening factor

    void `updateParametersInContext`(Context &*context*)¶
    :   Update the particle and screened pair parameters in a `Context` to match those stored in this `Force` object. This method provides an efficient method to update certain parameters in an existing `Context` without needing to reinitialize it. Simply call `setParticleParameters()` and `setScreenedPairParameters()` to modify this object’s parameters, then call `updateParametersInContext()` to copy them over to the `Context`.

        This method has several limitations. It can be used to modify the numeric parameters associated with a particle or screened pair (polarizability, thole, etc.), but not the identities of the particles they involve. It also cannot be used to add new particles or screenedPairs, only to change the parameters of existing ones.

    bool `usesPeriodicBoundaryConditions`() *const*¶
    :   Returns whether or not this force makes use of periodic boundary conditions.

        |  |  |
        | --- | --- |
        | Returns: | true if nonbondedMethod uses PBC and false otherwise |

High performance molecular simulation on GPUs


### Navigation

- Core Objects
- Forces
- Integrators
- Extras

©2015, Stanford University and the Authors.
|
Powered by Sphinx 1.3.1
& Alabaster 0.7.7
|
Page source
